# Physiological Responses of Two Submerged Macrophytes to Pulsed Aerial Exposure: Contrasting Photoprotection and Oxidative Defense

**DOI:** 10.3390/plants15213327

**Published:** 2026-10-27

**Authors:** Yanyi Zeng, Di Wu, Ru Zeng, Haiyan Li, Qianfu Liu, Wanling Yang, Yuan Gao, Lirong Bai, Chao Wang

**Affiliations:** 1 Pearl River Fisheries Research Institute, Chinese Academy of Fishery Sciences, Guangzhou 510380, China; 15697772728@163.com (D.W.); zengru000@163.com (R.Z.); lihaiyan@prfri.ac.cn (H.L.); liuqianfu@prfri.ac.cn (Q.L.); yangwanling@prfri.ac.cn (W.Y.); gaoyuan@prfri.ac.cn (Y.G.); 2 Fishery Ecological Environmental Monitoring Center of Pearl River Basin, Ministry of Agriculture and Rural Affairs, Guangzhou 510380, China; 3 Guangdong Provincial Key Laboratory of Aquatic Animal Immunology and Sustainable Aquaculture, Guangzhou 510380, China; 4 College of Marine Sciences, Beibu Gulf University, Qinzhou 535011, China; bailirong1152@163.com

**Keywords:** *Hydrilla verticillata*, *Vallisneria denseserrulata*, pulsed aerial exposure, photoprotection, oxidative defense, principal component analysis

## Abstract

Pulsed aerial exposure (PAE) is a major stressor for submerged macrophytes in tidal river networks, yet species-specific physiological mechanisms remain unclear. We conducted a 7-day PAE mesocosm experiment using *Hydrilla verticillata* (L.f.) Royle and *Vallisneria denseserrulata* (Makino) Makino to compare their photosynthetic performance, pigment retention, and oxidative defense. *Hydrilla* maintained lower baseline Y(II) and reliable EC_50_s (64.6–85.7 min/d). In contrast, *Vallisneria* showed higher baseline Y(II) but a transient Y(NPQ) increase peaking at 120 min/d that subsequently collapsed, greater pigment loss (total chlorophyll −68% vs. −44%), and highly uncertain or out-of-range EC_50_s. Although antioxidant enzyme activities were higher in *Vallisneria*, MDA accumulation was comparable between species. Principal component analysis (87.14% total variance) revealed that PC1 (42.03%) separated pigments from antioxidant enzymes, and PC2 (26.46%) captured photochemical efficiency. PC3 (18.65%) further indicated a transient oxidative burst and subsequent defense breakdown in *Vallisneria* under severe stress. These results suggest that *Hydrilla* exhibits a low light-capture strategy to mitigate PAE pressure, while *Vallisneria* exhibits an inducible defense that becomes overwhelmed. The quantitative parameters provide baseline data for macrophyte growth models, though field validation is required to confirm long-term ecological implications.

## 1. Introduction

Hydrological regimes are primary drivers of submerged macrophyte community assembly and succession. In dynamic water bodies such as rivers, floodplains, and tidal river networks, water-level fluctuations regulate light availability, gas exchange, and sediment properties, thereby shaping the distribution and diversity of submerged macrophytes [1,2]. In recent decades, however, widespread alteration of hydrological regimes—particularly the intensification of water-level fluctuations—has led to large-scale declines in submerged macrophyte communities worldwide [3,4,5]. Extreme droughts, such as the 2022 event in the Yangtze River basin, have further restructured these communities by suppressing clonal species [6]. Understanding how plants respond to water-level fluctuations is therefore not only a central question in aquatic ecology but also critical for predicting community change and guiding ecological restoration [7].

Tidal river networks are characterized by frequent and often large water-level fluctuations, making them one of the most dynamic freshwater ecosystems. Tidal action exposes submerged macrophytes in the drawdown zone to repeated aerial exposure lasting from tens of minutes to a few hours during each tidal cycle, a recurrent stressor termed pulsed aerial exposure (PAE). Unlike prolonged continuous flooding or extreme drought, PAE is high-frequency, short-duration, and recurrent, requiring plants to repeatedly adjust their physiological status during rapid dehydration–rehydration cycles [8,9,10]. Such events can cause photoinhibition and oxidative damage, disrupting the partitioning of absorbed light energy among photochemistry, regulated heat dissipation, and constitutive thermal dissipation [11,12]. In tropical tidal river networks such as the Pearl River Delta (PRD), upstream hydropower regulation superimposed on natural tides further intensifies the timing, frequency, and magnitude of PAE, particularly during the dry season (October–March) when submerged macrophytes initiate recovery, potentially pushing plants beyond their natural tolerance windows [13,14,15].

Field surveys in the PRD have revealed a striking phenomenon. *Vallisneria denseserrulata* (Makino) Makino was once the dominant species in multiple river sections but declined sharply and disappeared from most sections between 2015 and 2020 [4], whereas *Hydrilla verticillata* (L.f.) Royle persists as a common associated species [15]. A similar pattern has been reported in the tidal Potomac River, North America, where *H. verticillata* became locally dominant after the resurgence of submerged vegetation in 1983, while other areas supported mixed communities including *V. americana* [16,17]. This cross-regional consistency suggests that in tidal river networks with intensified PAE, *Hydrilla* may exhibit greater persistence than *Vallisneria*. Notably, these two species also exhibit sharply contrasting growth forms: *H. verticillata* produces erect, highly branched shoots with scaly leaves, whereas *V. denseserrulata* forms basal rosettes with long, flat leaves.

Such morphological differences may fundamentally influence how light is captured and how stress is endured. Plants can invest in constitutive defense, which is maintained regardless of stress, or in inducible defense, activated only after stress occurs [18,19]. These strategies differ in timing, cost, and reliability, and co-occurring species may favour one over the other [20]. We therefore hypothesize that *H. verticillata* may rely on a constitutive strategy, maintaining low light capture and high thermal dissipation before stress occurs, whereas *V. denseserrulata* may depend on an inducible strategy that becomes exhausted under recurrent exposure. This hypothesis, however, has not been systematically tested under PAE. Early desiccation studies primarily focused on acute, single-event stress and lacked mechanistic insights into photosynthesis or antioxidant responses [8,21,22]. Although recent work has confirmed a negative relationship between desiccation duration and plant viability [9,10], the integration of photochemical energy allocation (Y(II), Y(NPQ), and Y(NO)) with oxidative defense mechanisms in submerged plants under recurrent PAE remains largely unexplored.

To test this hypothesis, we conducted a 7-day PAE mesocosm experiment using whole plants of *H. verticillata* and *V. denseserrulata*, two submerged macrophytes with contrasting leaf morphologies. We aimed to: (1) quantify their physiological response strategies and tolerance thresholds to PAE; (2) characterize the physiological basis of their differences in light energy allocation and oxidative defense; and (3) provide species-specific parameters for submerged macrophyte growth models in tidal river networks.

## 2. Results

### 2.1. Chlorophyll Fluorescence Parameters and Energy Allocation

**Figure 1:**
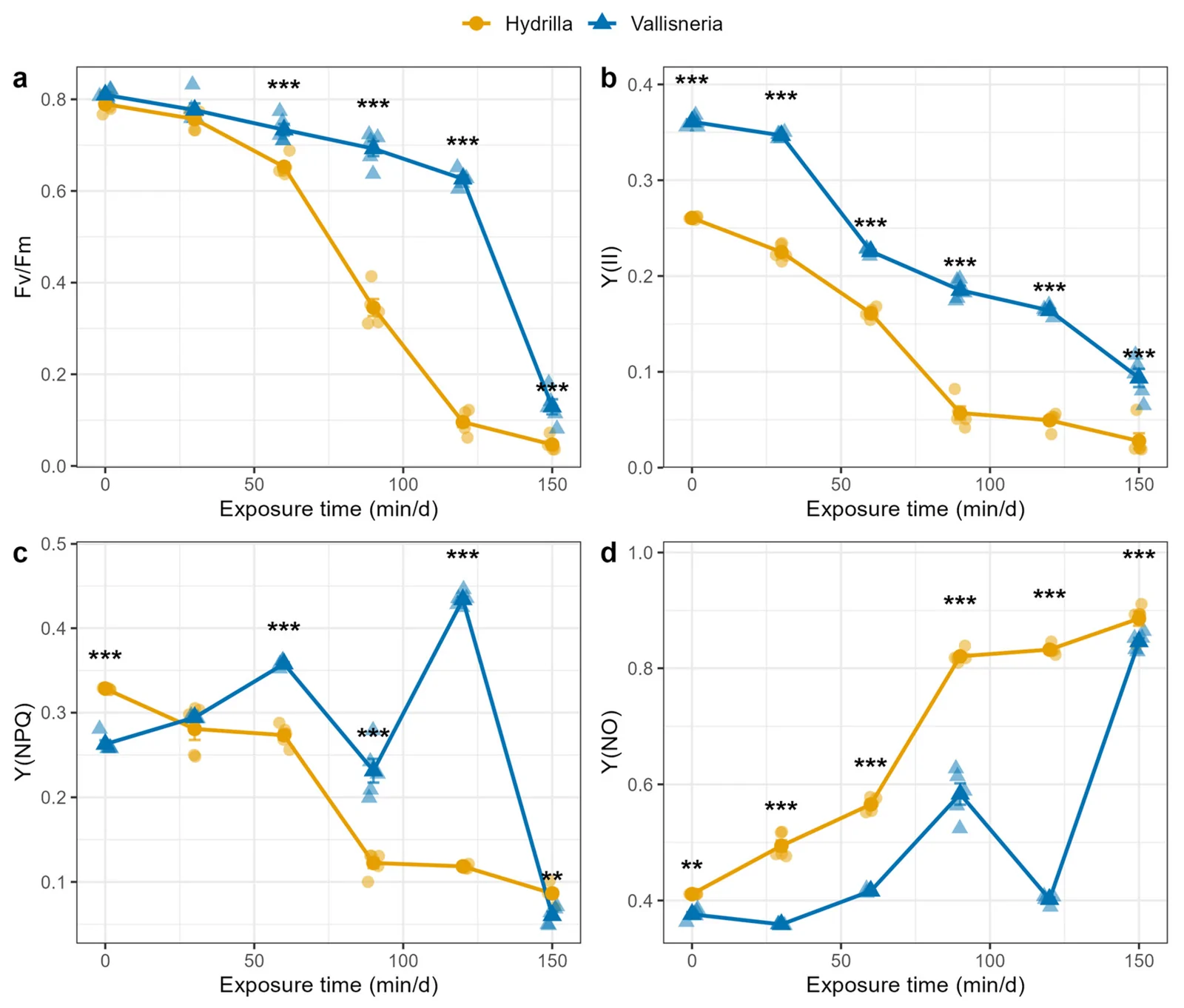
Chlorophyll fluorescence parameters Fv/Fm (**a**), Y(II) (**b**), Y(NPQ) (**c**), and Y(NO) (**d**) in *Hydrilla* (orange circles) and *Vallisneria* (blue triangles) under increasing PAE durations (0–150 min/d). Each symbol represents an individual replicate (*n* = 5 per treatment); symbols with error bars indicate mean ± SE. Asterisks indicate significant interspecific differences at each exposure time (two-way ANOVA + FDR; ** *p* < 0.01, *** *p* < 0.001).

Chlorophyll fluorescence parameters under increasing PAE (0–150 min/d) are shown in Figure 1. Two-way ANOVA showed significant species × treatment interactions for Fv/Fm, Y(II), Y(NPQ) and Y(NO) (all *p* < 0.001; Table 1). Fv/Fm remained relatively stable in both species below 60 min/d, but diverged sharply at higher intensities (Figure 1a). Y(II) decreased with increasing PAE in both species, with *Vallisneria* maintaining higher values than *Hydrilla* across all exposure durations (Figure 1b). Y(NPQ) in *Hydrilla* showed a gradual decline from the control onwards; in *Vallisneria*, it increased initially, peaking at 120 min/d, before declining sharply (Figure 1c). Y(NO) increased progressively with PAE in *Hydrilla*, reaching 0.89 at 150 min/d; in *Vallisneria*, it rose up to 90 min/d, then declined transiently at 120 min/d, before rising sharply to 0.85 at 150 min/d (Figure 1d). *Hydrilla* showed lower baseline Y(II) and higher Y(NO), whereas *Vallisneria* showed higher baseline Y(II) and a transient increase in Y(NPQ).

### 2.2. Photosynthetic Pigment Responses

**Figure 2:**
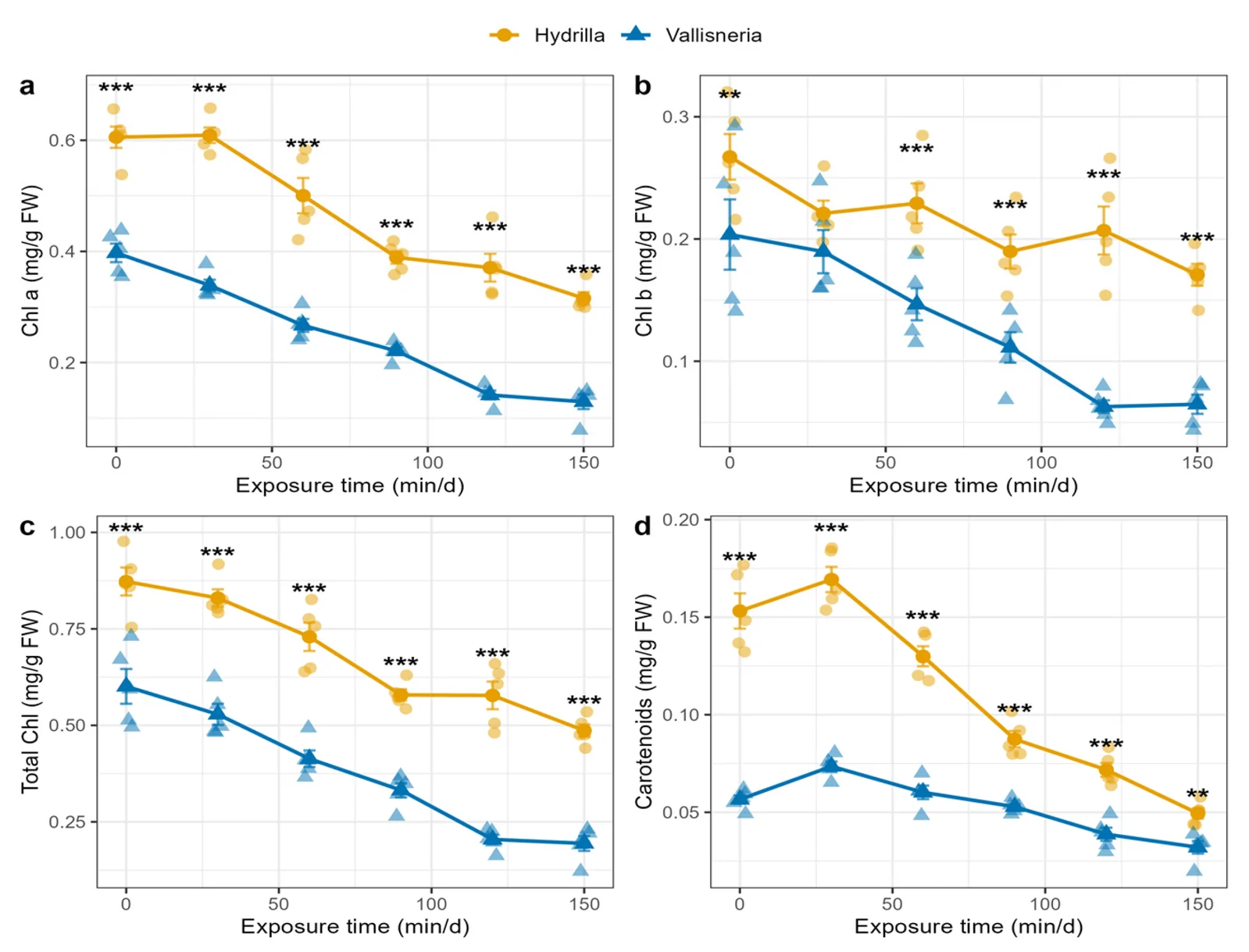
Photosynthetic pigment contents—chlorophyll a (**a**), chlorophyll b (**b**), total chlorophyll (**c**), and carotenoids (**d**)—in *Hydrilla* (orange circles) and *Vallisneria* (blue triangles) under increasing PAE durations (0–150 min/d). Each symbol represents an individual replicate (*n* = 5 per treatment); symbols with error bars indicate mean ± SE. Asterisks indicate significant interspecific differences at each treatment level (two-way ANOVA + FDR; ** *p* < 0.01, *** *p* < 0.001).

All measured pigments decreased with increasing PAE in both species, with *Hydrilla* maintaining significantly higher concentrations than *Vallisneria* across all treatments (Figure 2). Two-way ANOVA showed significant species × treatment interactions for Chl a, Chl b and carotenoids, but not for total chlorophyll (Table 1). This non-significant interaction indicates that the overall decline in total chlorophyll followed a similar pattern in both species. At 150 min/d, total chlorophyll declined by 68% (from an average of 0.60 to 0.19 mg/g FW) in *Vallisneria*, compared to 44% (from 0.87 to 0.48 mg/g FW) in *Hydrilla*. For carotenoids, *Hydrilla* showed a 68% reduction (from 0.15 to 0.05 mg/g FW), whereas *Vallisneria* declined by 43% (from 0.06 to 0.03 mg/g FW) from an already low baseline. Consequently, despite the larger relative decline, the absolute carotenoid concentration remaining in *Hydrilla* at 150 min/d (0.05 mg/g FW) was still higher than that in *Vallisneria* (0.03 mg/g FW).

### 2.3. Exposure Time–Response Relationships

**Figure 3:**
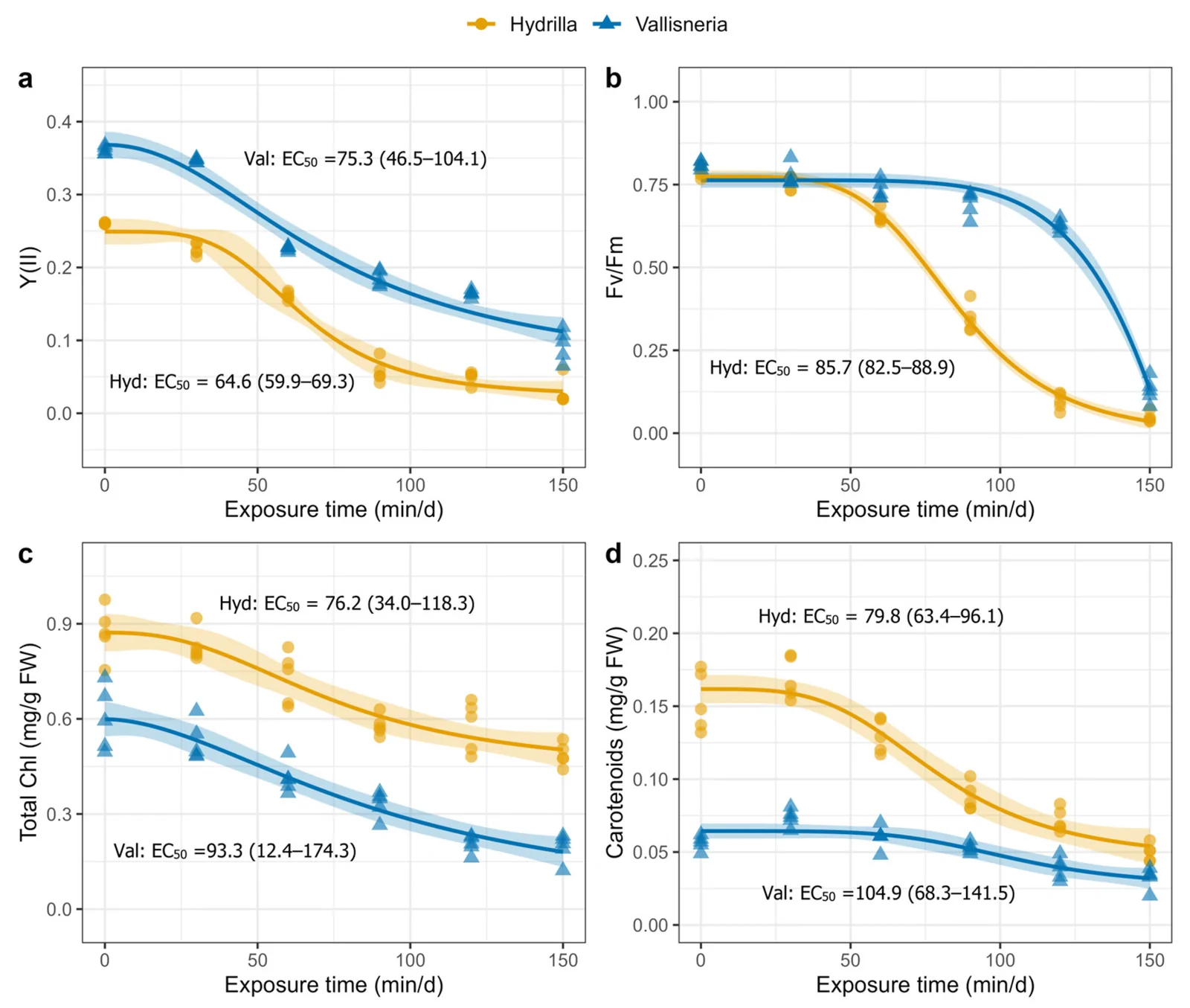
Exposure time–response curves for Y(II) (**a**), Fv/Fm (**b**), total chlorophyll (**c**), and carotenoids (**d**) in *Hydrilla* (orange circles) and *Vallisneria* (blue triangles) under 0–150 min/d PAE. Each symbol represents an individual replicate (*n* = 5 per treatment). Solid lines: LL.4 fits; shaded ribbons: 95% CI. EC_50_ (95% CI) are annotated for reliable estimates.

Exposure time–response curves for Y(II), Fv/Fm, total chlorophyll, and carotenoids under 0–150 min/d PAE are shown in Figure 3. Model validation indicated that the four-parameter log-logistic (LL.4) model provided an excellent fit for all parameters, with Pseudo-R^2^ values ranging from 0.807 to 0.992 (Table S1). In *Hydrilla*, reliable EC_50_ estimates with relatively narrow 95% CIs were obtained across all parameters: Y(II) (64.6 min/d, 95% CI: 59.9–69.3), total chlorophyll (76.2 min/d, 95% CI: 34.0–118.3), carotenoids (79.8 min/d, 95% CI: 63.4–96.1), and Fv/Fm (85.7 min/d, 95% CI: 82.5–88.9). In contrast, *Vallisneria* exhibited highly uncertain threshold estimates. The EC_50_ for Fv/Fm in *Vallisneria* was mathematically extrapolated to 211.6 min/d (95% CI: 121.0–302.3 min/d), which far exceeded the maximum experimental exposure duration (150 min/d); therefore, this out-of-range estimate is reported in Table S1 but not annotated in Figure 3. Although the model yielded EC_50_ estimates within the tested range for Y(II) (75.3 min/d, 95% CI: 46.5–104.1), total chlorophyll (93.3 min/d, 95% CI: 12.4–174.3), and carotenoids (104.9 min/d, 95% CI: 68.3–141.5), their 95% CIs were substantially wider than those of *Hydrilla*, indicating a high degree of uncertainty.

Direct interspecies comparisons based on the 95% CIs revealed that the EC_50_ of Fv/Fm was significantly lower in *Hydrilla* than in *Vallisneria*, as their confidence intervals did not overlap. However, for Y(II), total chlorophyll, and carotenoids, the 95% CIs overlapped between the two species, indicating no statistically significant differences in their dose–response thresholds for these specific parameters.

### 2.4. Antioxidant Enzyme Activities and Lipid Peroxidation

**Figure 4:**
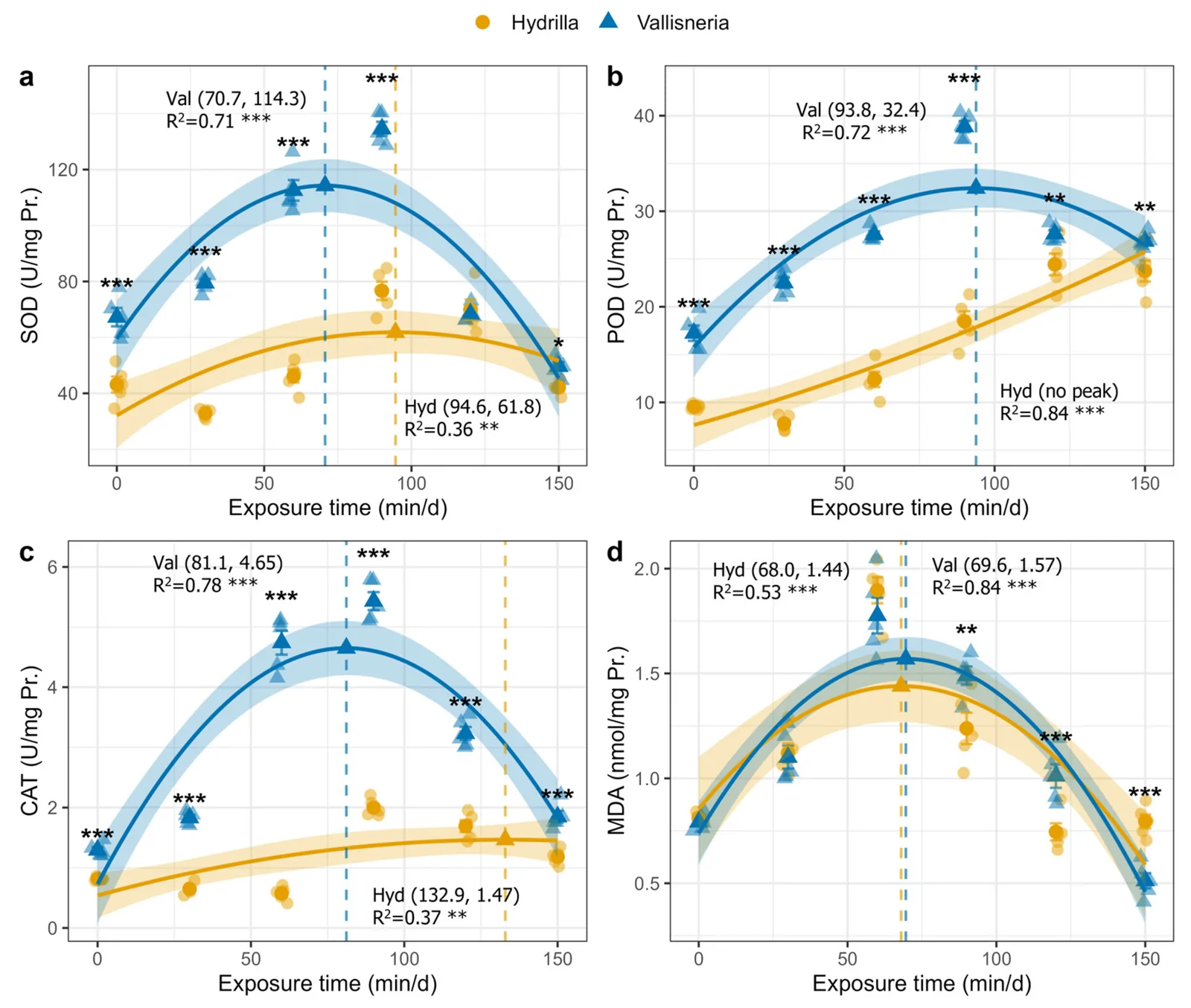
Antioxidant-related responses—SOD (**a**), POD (**b**), CAT (**c**), and MDA (**d**) of *Hydrilla* (orange circles) and *Vallisneria* (blue triangles) under 0–150 min/d PAE. Each symbol represents an individual replicate (*n* = 5 per treatment). Solid lines: quadratic fits; shaded ribbons: 95% CI. Dashed lines and triangles indicate peak dose and peak value, respectively. Asterisks indicate significant interspecific differences (two-way ANOVA + FDR; * *p* < 0.05, ** *p* < 0.01, *** *p* < 0.001).

Antioxidant enzyme activities and MDA contents are shown in Figure 4. Two-way ANOVA revealed significant species × treatment interactions for SOD, POD, CAT, and MDA (all *p* < 0.001; Table 1). *Vallisneria* exhibited significantly higher activities of SOD, POD and CAT than *Hydrilla* across the exposure gradient (all *p* < 0.001 for species effect).

Quadratic polynomial models revealed distinct temporal dynamics between the two species. In *Vallisneria*, the activities of SOD (peak at 70.7 min/d, 114.3 U/mg protein), CAT (peak at 81.1 min/d, 4.65 U/mg protein), and POD (peak at 93.8 min/d, 32.4 U/mg protein) exhibited strong bell-shaped responses (*R*^2^ = 0.71–0.78, all *p* < 0.001). Notably, the SOD peak (70.7 min/d) tightly coincided with the MDA peak (69.6 min/d, 1.57 nmol/mg protein). In contrast, *Hydrilla* exhibited a delayed response pattern. Its MDA peaked earlier at 68.0 min/d (1.44 nmol/mg protein), whereas the peaks of SOD (94.6 min/d, 61.8 U/mg protein, *R*^2^ = 0.36, *p* < 0.01) and CAT (132.9 min/d, 1.47 U/mg protein, *R*^2^ = 0.37, *p* < 0.01) were substantially delayed. Furthermore, POD increased monotonically without reaching a clear peak (*R*^2^ = 0.84, *p* < 0.001). Overall, the MDA peak preceded the major antioxidant enzyme peaks in *Hydrilla*, whereas the SOD peak tightly coincided with the MDA peak in *Vallisneria*.

### 2.5. Integrated Physiological Responses

**Figure 5:**
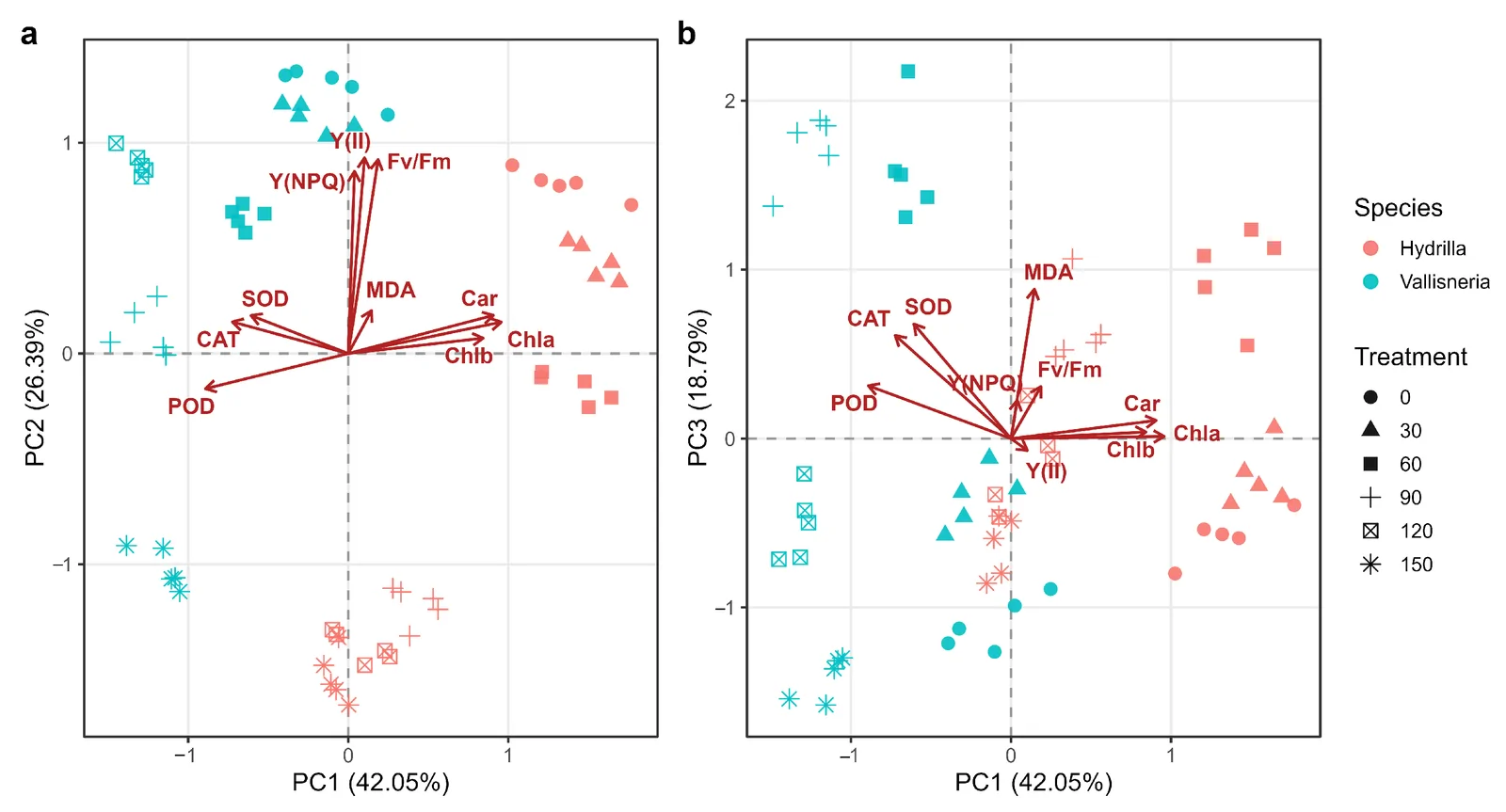
Principal component analysis (PCA) biplots of 10 physiological parameters in *Hydrilla* and *Vallisneria* under increasing PAE (0–150 min/d). (**a**) PC1 vs. PC2. (**b**) PC1 vs. PC3. Each point represents one replicate; points are colored by species and shaped by treatment. Red arrows indicate variable loadings, where the direction and length represent the contribution of each parameter to the principal components.

Principal component analysis (PCA) was performed on the 10 physiological variables to assess whether the two species occupied distinct physiological spaces under PAE (Figure 5). The data met the assumptions for PCA (KMO = 0.703; Bartlett’s test of sphericity: χ^2^ = 734.05, *df* = 45, *p* < 0.001), and the first three principal components explained 87.14% of the total variance. PC1 (42.03%) primarily separated photosynthetic pigments (Chl a, Chl b, and Car; positive loadings) from antioxidant enzymes (POD, SOD, and CAT; negative loadings). PC2 (26.46%) represented photochemical parameters, with positive loadings for Fv/Fm, Y(II), and Y(NPQ). PC3 (18.65%) captured the dynamics of oxidative stress, with high positive loadings for MDA, SOD, and CAT.

Samples from the control and low-PAE treatments of *Vallisneria* (0–30 min/d) clustered in the positive PC2 region, characterized by high photochemical efficiency. Under moderate-to-high PAE (60–90 min/d), *Vallisneria* samples shifted to the extreme negative region of PC1 (Figure 5a) and the positive region of PC3 (Figure 5b), indicating a peak in oxidative stress (MDA) and antioxidant enzyme activities (SOD and CAT). At 120 min/d, *Vallisneria* showed a sharp decline in PC3 into the negative region while remaining in the positive PC2 region. By 150 min/d, it had shifted sharply into the negative regions of both PC2 and PC3. However, *Hydrilla* under control and low-to-moderate PAE (0–60 min/d) mainly clustered in the positive PC1 region, reflecting higher pigment concentrations. At the highest PAE (90–150 min/d), *Hydrilla* shifted towards the negative PC2 region, indicating a decline in photochemical efficiency. However, *Hydrilla* exhibited minimal fluctuations along PC3 across all treatments compared to the dramatic shifts observed in *Vallisneria*. These distribution patterns indicate that the two species occupied distinct physiological spaces under increasing PAE.

## 3. Discussion

### 3.1. Photoprotection and Pigment Dynamics: Constitutive Versus Inducible Defense

Within the classic Lake model framework of chlorophyll fluorescence [23,24], absorbed light energy is partitioned into three pathways: actual photochemical efficiency (Y(II)), regulated photoprotective energy dissipation (Y(NPQ)), and non-regulated energy dissipation (Y(NO)). Y(NPQ) represents an active, inducible defense mechanism (e.g., the xanthophyll cycle) that safely dissipates excess energy, whereas Y(NO) represents passive thermal dissipation and indicates the failure of photoprotection or potential photodamage [12,23,24]. The contrasting patterns in these three parameters between the two species point to a fundamental difference in the timing of defense activation.

*Hydrilla* maintained lower baseline Y(II), a higher baseline Y(NO), and showed a gradual decline in Y(NPQ) from the control onwards. The elevated Y(NO) in *Hydrilla* primarily reflects its constitutively low light capture (low Y(II)), rather than an active protective mechanism. Instead, this species employs a pre-emptive strategy by constitutively down-regulating light capture to limit excitation pressure, thereby avoiding the need for rapid photoprotective upregulation. In contrast, *Vallisneria* maintained higher baseline Y(II) and showed a transient increase in Y(NPQ), peaking at 120 min/d before declining sharply. This pattern is characteristic of a reactive strategy, in which light capture is maximized under favorable conditions, and active photoprotection (Y(NPQ)) is induced only after stress arrives, with capacity rapidly exhausted and eventually replaced by an uncontrolled rise in Y(NO) [12].

This functional divergence was structurally underpinned by differential pigment retention. *Hydrilla* maintained significantly higher Chl a, Chl b, total chlorophyll, and carotenoid concentrations across PAE treatments, supporting the structural integrity of its light-harvesting apparatus and enabling reliable EC_50_ thresholds. In contrast, *Vallisneria* suffered accelerated pigment degradation, particularly of carotenoids, which corresponded with the collapse of Y(NPQ) capacity and the rise of Y(NO). Because carotenoids serve dual roles as light-harvesting pigments and NPQ cofactors, their loss may create a positive feedback loop in which pigment degradation reduces NPQ capacity and consequently increases excitation pressure, thereby accelerating further photo-oxidative damage [12]. This interpretation is consistent with the broader pattern that induced defenses, while potentially strong, are inherently less reliable than constitutive ones when stress is sustained [25].

Comparative evidence from diverse aquatic photoautotrophs supports the generality of this distinction. In intertidal seagrasses, the tolerant species *Halophila beccarii* enhances NPQ and cyclic electron flow to dissipate excess energy during air exposure, whereas sensitive species lack such sustained photoprotective capacity [26]. Similarly, in the red alga *Neoporphyra haitanensis*, desiccation tolerance is associated with coordinated upregulation of antioxidants and LEA proteins. This constitutive preparedness closely mirrors the pre-emptive strategy of *Hydrilla* [27]. Macroalgal studies further demonstrate that tolerant stages of *Pyropia yezoensis* exhibit well-defined thermal and desiccation optima with quantifiable thresholds, while sensitive life-history stages, such as those of *Caulerpa lentillifera*, show unreliable thresholds and fail to recover after moderate stress [28,29]. Together, these observations confirm the photoprotection efficiency and threshold reliability, rather than photoprotective capacity alone, consistently separate tolerant from sensitive strategies across phylogenetically distant lineages. In fluctuating environments, preparation outperforms reaction.

### 3.2. Oxidative Defense: Investment Alone Is Insufficient

*Vallisneria* induced SOD, POD, and CAT at levels far exceeding those of *Hydrilla* across the exposure gradient, yet MDA accumulation remained comparable between species. This high-investment but limited-protection pattern reveals a defense efficiency bottleneck, indicating a decoupling between enzyme induction and actual ROS scavenging. Efficient ROS scavenging is not solely dependent on enzyme abundance [30], but rather requires a highly coordinated network of enzymatic and non-enzymatic antioxidants to facilitate cellular protection and subsequent physiological repair [31]. Simply mounting higher enzyme activities, without corresponding increases in scavenging efficiency or substrate availability, yields diminishing returns [25,32].

The temporal dynamics of these biomarkers further support this decoupling. In *Vallisneria*, the peaks of SOD and CAT tightly coincided with the MDA peak, whereas in *Hydrilla*, the MDA peak clearly preceded the major enzyme peaks. This suggests that the basal mechanisms of *Hydrilla* were sufficient to limit the initial oxidative burst, avoiding the need for an overwhelming enzymatic response. This inefficiency pattern is consistent with previous findings on *Vallisneria* under other stress contexts. Under bisphenol A, nanoplastics combined with cadmium, and PFAS, *Vallisneria* consistently exhibits high enzyme induction yet persistent oxidative damage [33,34,35,36]. *Hydrilla*, by contrast, maintains more coordinated defense, including antagonistic masking of oxidative damage under combined PFAS and microplastic stress [37], implying superior ROS buffering capacity that operates independently of enzyme level. The moderate investment–high efficiency strategy of *Hydrilla* suggests that an optimal defense investment level exists, beyond which additional enzyme induction yields no proportional benefit.

### 3.3. Integrated Physiological Responses: Linking Photoprotection to Oxidative Defense

The multivariate PCA provided an integrated view of how the two species partitioned their physiological space under PAE. *Vallisneria* followed a dynamic trajectory, transitioning from a state dominated by photochemical performance (PC2) to a synchronized peak of oxidative damage and antioxidant enzymes (PC3), before undergoing a comprehensive collapse at extreme stress. In contrast, *Hydrilla* maintained a relatively constrained trajectory along these axes, reflecting a more stable physiological baseline. This multidimensional divergence aligns with the widely recognized trade-off between photosynthetic capacity and stress tolerance in plant ecology [18,38].

A possible structural basis for this strategy lies in the species’ morphology. Unlike *Vallisneria*, both stems and leaves of erect-type *Hydrilla* are photosynthetically active, with the stem considered crucial for stress acclimation [39]. Furthermore, *Hydrilla* allocates a high proportion of its biomass to aboveground shoots [40], and its stem fragments can rapidly resume protein and carbohydrate synthesis upon rehydration to produce new shoots [41,42]. These morphological and regenerative traits may collectively buffer against localized leaf damage during PAE, allowing *Hydrilla* to sustain its physiological functions under recurrent stress.

These integrated findings have implications for understanding the field distribution of these species. While *V. natans* is known to be highly competitive under stable, low-light conditions due to its low light compensation point [43], light availability is likely not the primary limiting factor for *Vallisneria* in PAE-dominated tidal river networks. Instead, the physiological stability observed in *Hydrilla* may better explain its persistence alongside the decline of *Vallisneria*. However, because our data do not directly assess long-term recovery capacity, these inferences remain limited to short-term physiological responses and require further field validation.

### 3.4. Limitations and Future Perspectives

As a mesocosm simulation, the fixed midday exposure and 7-day duration do not fully capture natural tidal stochasticity, and the single 24 h recovery measurement reflects residual rather than immediate stress responses. Furthermore, because stem and leaf tissues were not physically separated in *Hydrilla*, the direct contribution of its photosynthetic stem to stress buffering remains untested. Our conclusions are therefore limited to the physiological responses of two species under controlled conditions. To draw definitive conclusions regarding long-term population persistence, future research must extend beyond short-term physiological assays to encompass full life-cycle studies, tracking growth, reproduction, and overwintering survival under natural conditions. Additionally, future work should incorporate multiple sampling times throughout the dehydration–rehydration cycles, physically separate stem and leaf tissues, and validate the proposed physiological trade-off across a broader range of submerged macrophytes under natural field conditions. To further resolve the molecular mechanisms underlying the contrasting strategies, integrated transcriptomic and metabolomic analyses could identify key regulatory pathways and candidate genes or metabolites that distinguish constitutive from inducible defense.

## 4. Materials and Methods

### 4.1. Plant Materials and Acclimation

The experiment was conducted at the Pearl River Fisheries Research Institute (Guangzhou, China; 23°4′7″ N, 113°13′3″ E) from February to March 2025 in an outdoor pond under natural sunlight, simulating the spring recovery and growth period of submerged macrophytes. *Hydrilla verticillata* (L.f.) Royle (hereafter *Hydrilla*) and *Vallisneria denseserrulata* (Makino) Makino (hereafter *Vallisneria*) were obtained from outdoor stock cultures. Healthy *Vallisneria* plants were trimmed to 20 cm (7–8 leaves, 5 cm roots); 25 cm multi-branched apical segments of *Hydrilla* were prepared with the lower 5 cm defoliated. *Vallisneria* plants and *Hydrilla* apical segments were planted separately in plastic pots (20 cm diameter, 10 cm sediment depth; seven individuals per pot). The roots of *Vallisneria* and the lower 5 cm of *Hydrilla* segments were inserted into the sediment, so that the aboveground parts of both species were approximately 20 cm in height. Sediment was collected from the same outdoor pond and passed through a 2 mm sieve before use. All pots were placed in submerged net cages in the same outdoor pond for two weeks of acclimation before the experiment.

### 4.2. Experimental Design

The experiment followed a two-factor completely randomized design, with species and PAE duration as the two fixed factors. For each species, 30 pots were randomly allocated to six treatments: control (0 min/d) and five stress groups (30, 60, 90, 120, 150 min/d), covering a gradient from mild to severe PAE intensity. Each treatment had five replicate pots, with each pot as an independent experimental unit. All pots were randomly arranged within the submerged net cages to minimize positional effects.

**Figure 6:**
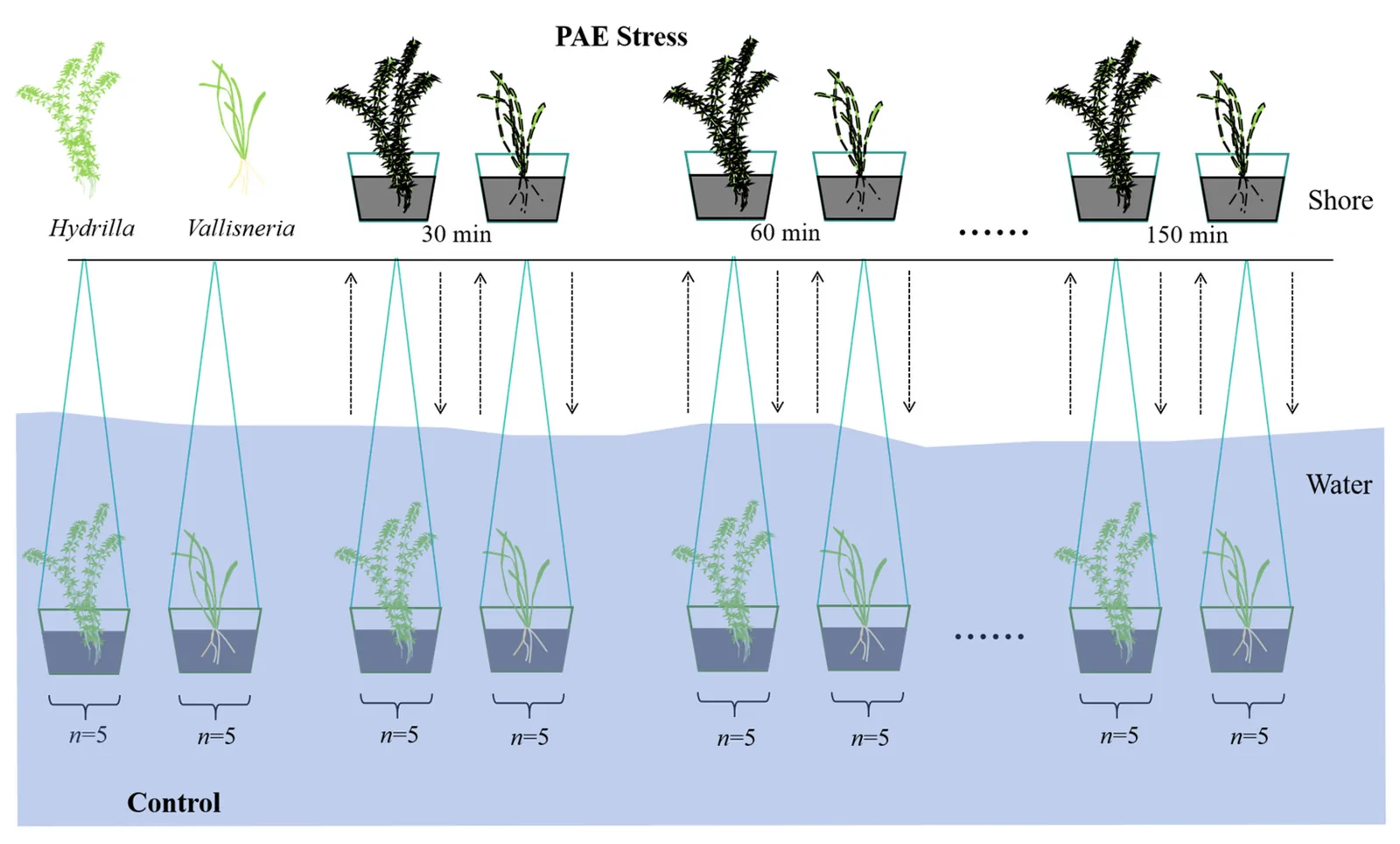
Schematic diagram of the 7-day pulsed aerial exposure (PAE) mesocosm experiment. Pots were acclimated in submerged net cages. During each daily exposure, stress pots were lifted from the water to the shore, exposed to air for the prescribed duration, and then returned to the water; control pots remained submerged throughout the experiment.

For seven consecutive days, stress pots were lifted out of the water at midday, exposing the plants completely to air, and returned to the water after the prescribed duration; control pots remained submerged throughout (Figure 6). Midday was selected to standardize the PAE treatment. In tropical and subtropical tidal river networks, midday typically features high irradiance, elevated air temperature, and low humidity, making it a representative and relatively severe period of PAE stress. Due to mixed semidiurnal tides, natural low-tide times shift daily; therefore, a fixed midday exposure was used to standardize treatment conditions rather than to precisely replicate the natural tidal rhythm. During the 7-day PAE experiment, midday environmental conditions were as follows: air temperature 25.3 ± 3.1 °C, photosynthetic photon flux density (PPFD) 335 ± 102 μmol/m^2^/s, water temperature 19.9 ± 1.1 °C, pH 8.03 ± 0.21, dissolved oxygen 7.97 ± 1.25 mg/L, redox potential 170 ± 30 mV, and conductivity 134 ± 7 μS/cm.

After the final exposure on day 7, plants were allowed to recover in water for 24 h before sampling to assess the residual effects of PAE after a standardized recovery interval. Chlorophyll fluorescence was measured in situ on a randomly selected plant per pot. After fluorescence measurement, all plants from the same pot were harvested, and the aboveground parts were pooled as one composite sample for subsequent pigment and biochemical analyses. Specifically, for *Vallisneria*, several fully expanded leaves were randomly selected; for *Hydrilla*, the entire aboveground part (stems with leaves) was harvested because both stems and leaves are photosynthetically active. Thus, both fluorescence and biochemical measurements were derived from the same experimental unit (pot), with the pot as the independent replicate (*n* = 5). The randomly selected plant for fluorescence measurement was assumed to represent the physiological status of that pot.

### 4.3. Chlorophyll Fluorescence Measurement

After 20 min dark adaptation, the maximum quantum yield of PSII (Fv/Fm) was measured using a portable chlorophyll fluorometer (Diving-PAM-II; Heinz Walz GmbH, Effeltrich, Germany). Under actinic light (400 μmol/m^2^/s), steady-state fluorescence (Fs), light-adapted maximum fluorescence (Fm′), and light-adapted minimum fluorescence (Fo′) were recorded. The instrument automatically calculated the effective quantum yield of PSII photochemistry (Y(II)), quantum yield of regulated energy dissipation (Y(NPQ)), and quantum yield of non-regulated energy dissipation (Y(NO)) based on the Lake model [23,24], satisfying Y(II) + Y(NPQ) + Y(NO) = 1. All parameters were exported via WinControl-3.

### 4.4. Photosynthetic Pigment Determination

Fresh leaf (and stem) samples (ca. 0.1 g) were extracted in 10 mL of acetone–ethanol mixture (1:1, *v*/*v*) in the dark for 24 h. Absorbance of the extract was measured at 663 nm, 646 nm, and 470 nm using a UV-Vis spectrophotometer (UV2700; Shimadzu, Kyoto, Japan). Concentrations of chlorophyll a (Chl a), chlorophyll b (Chl b), and carotenoids (Car) were calculated as follows [44,45]:

Chl a (mg/g FW) = 12.21 × A_663_ − 2.81 × A_646_, (1)

Chl b (mg/g FW) = 20.13 × A_646_ − 5.03 × A_663_ (2)

Car (mg/g FW) = (1000 × A_470_ − 3.27 × Chl a − 104 × Chl b)/229 (3)

Total chlorophyll was calculated as the sum of Chl a and Chl b.

### 4.5. Antioxidant Enzyme Activity and Lipid Peroxidation Assays

Fresh leaf (and stem) samples were immediately snap-frozen in liquid nitrogen and stored at −80 °C until analysis to minimize enzymatic degradation. For enzyme extraction, frozen tissues (ca. 0.2 g) were ground to a fine powder in liquid nitrogen using a pre-chilled mortar and pestle, and then homogenized in 50 mM phosphate buffer (pH 7.8). The homogenate was centrifuged at 12,000× *g* for 20 min at 4 °C, and the supernatant was used for enzyme activity assays. All extraction procedures were performed strictly on ice or at 4 °C. Commercial kits (Nanjing Jiancheng Bioengineering Institute, Nanjing, China) were used following the manufacturer’s protocols: superoxide dismutase (SOD, EC 1.15.1.1) by the NBT method, peroxidase (POD, EC 1.11.1.7) by the guaiacol method, catalase (CAT, EC 1.11.1.6) by UV absorption, and malondialdehyde (MDA) by the TBA method.

Protein content was determined using a Bradford protein assay kit (Nanjing Jiancheng Bioengineering Institute, Nanjing, China) with bovine serum albumin as the standard. One unit (U) of SOD activity was defined as the amount of enzyme required to inhibit NBT photoreduction by 50% per minute. One unit of POD activity was defined as the amount of enzyme that catalyzed the oxidation of 1 μg guaiacol per minute. One unit of CAT activity was defined as the amount of enzyme that decomposed 1 μmol H_2_O_2_ per minute. Enzyme activities were expressed as U/mg protein, and MDA content was expressed as nmol/mg protein. All assays were read on a microplate reader (Bio Tek Cytation 5, Winooski, VT, USA).

### 4.6. Data Analysis and Statistics

All data were analyzed with the pot as the independent replicate. Data were first checked for normality (Shapiro–Wilk test) and homogeneity of variances (Levene’s test). Unequal variances were handled using Welch’s *t*-test. All statistical tests were two-tailed with α = 0.05. Two-way ANOVA, dose–response curve fitting, and data visualization were performed in R version 4.3.5 (R Foundation for Statistical Computing, Vienna, Austria), while principal component analysis (PCA) was conducted in SPSS 20.0 (IBM, Armonk, NY, USA).

Two-way ANOVA (species × treatment) was conducted for all physiological variables using the *aov()* function in R, based on the completely randomized design. When the interaction was significant, simple-effects analysis (*t*-tests between species within each treatment) was performed using the *emmeans* package, followed by FDR correction.

Dose–response curves for photosynthetic parameters were fitted with a four-parameter log-logistic model (LL.4) using the *drc* package in R. Model goodness-of-fit was assessed using the coefficient of determination (Pseudo-R^2^). EC_50_ values and their 95% confidence intervals (CIs) were estimated via the delta method. Direct interspecies comparisons of EC_50_ sensitivity were evaluated by assessing the overlap of the 95% CIs. For oxidative stress parameters, quadratic polynomial models were fitted against exposure time for each species separately; peak dose and response were derived from the vertex when the quadratic coefficient was negative.

For PCA, 10 standardized physiological variables were analyzed: Y(II), Y(NPQ), Fv/Fm, Chl a, Chl b, Car, POD, SOD, CAT, and MDA. Note that Y(NO) and total chlorophyll were excluded from the PCA to avoid mathematical collinearity (since Y(II) + Y(NPQ) + Y(NO) = 1, and total chlorophyll = Chl a + Chl b). The 10 variables were analyzed in SPSS using the principal component extraction method with Varimax rotation. The factor scores and rotated component matrix were exported from SPSS. The first three principal components were extracted, and the PCA biplot was generated using the *ggplot2* package in R to visualize the integrated physiological responses.

## 5. Conclusions

This study compared the physiological responses of *Hydrilla verticillata* and *Vallisneria denseserrulata* to a 7-day PAE mesocosm treatment, revealing contrasting strategies between the two species. *Hydrilla* maintained a lower baseline Y(II), stable pigment retention and reliable EC_50_ thresholds; its higher baseline Y(NO) primarily reflected constitutively low light capture rather than an active protective mechanism. *Vallisneria* showed a transient Y(NPQ) increase that subsequently collapsed, greater pigment loss, and unreliable EC_50_ thresholds. Despite its higher antioxidant enzyme activities, MDA accumulation remained comparable, indicating a bottleneck in defense efficiency. The integrated PCA further revealed that the two species occupied distinct physiological spaces under increasing PAE. These results suggest that *Hydrilla* relies more on constitutive protection, whereas *Vallisneria* depends on inducible defense that becomes exhausted under severe recurrent PAE. The EC_50_ and photoprotective parameters provide useful baseline inputs for macrophyte growth models, but full life-cycle studies and field validation are needed to determine their long-term ecological consequences.

## Figures and Tables

**Table 1:** Two-way ANOVA results (F-values and significance) for physiological parameters under Species × Treatment.

Parameter Category	Parameter	Species	Treatment	Species × Treatment
Photosynthetic performance	Y(II)	1312.5 ***	916.7 ***	17.34 ***
	Y(NPQ)	358.6 ***	408.5 ***	215.0 ***
	Y(NO)	1469.3 ***	1099.1 ***	182.5 ***
	Fv/Fm	666.9 ***	1039.5 ***	151.7 ***
Photosynthetic pigment content	Chl a	518.6 ***	98.2 ***	2.4 *
	Chl b	87.3 ***	16.6 ***	3.0 *
	Total Chl	361.1 ***	68.1 ***	1.2 ns
	Car	509.5 ***	97.0 ***	29.8 ***
Oxidative stress & antioxidant defense	POD	683.1 ***	154.4 ***	50.0 ***
	SOD	537.4 ***	149.2 ***	61.4 ***
	CAT	1203.3 ***	229.5 ***	128.4 ***
	MDA	0.2 ns	1447.7 ***	8.7 ***

Note: * *p* < 0.05, *** *p* < 0.001, ns not significant.

## Data Availability

The data presented in this study are available in the Supplementary Materials. Further inquiries can be directed to the corresponding author.

## Supplementary Materials

The following supporting information can be downloaded at https://www.mdpi.com/article/10.3390/plants15213327/s1. Table S1: EC50s estimates; Table S2: Rotated component matrix of PCA; Table S3: Principal component scores (PC1, PC2, and PC3); Table S4: Compiled raw data.
