# Supplementary material for: Physiological Responses of Two Submerged Macrophytes to Pulsed Aerial Exposure: Contrasting Photoprotection and Oxidative Defense
Source: Plants (Basel). 2026 Oct 27;15(21):3327. doi: 10.3390/plants15213327 (PMC13765662; doi:10.3390/plants15213327)
Supplement: Supplementary file 1 [file plants-15-03327-s001.zip › Table S1 EC50s estimates.pdf]

Table S1. Model validation and EC_{50} estimates for photosynthetic parameters under PAE.

Species	Variable	EC_{50} (min/d)	95% CI Lower (min/d)	95% CI Upper (min/d)	Pseudo-R^2
Hydrilla verticillata	Y(II)	64.6	59.9	69.3	0.958
Hydrilla verticillata	Fv/Fm	85.7	82.5	88.9	0.992
Hydrilla verticillata	Total chlorophyll	76.2	34.0	118.3	0.807
Hydrilla verticillata	Carotenoids	79.8	63.4	96.1	0.951
Vallisneria denseserrulata	Y(II)	75.3	46.5	104.1	0.952
Vallisneria denseserrulata	Fv/Fm	211.6	121.0	302.3	0.975
Vallisneria denseserrulata	Total chlorophyll	93.3	12.4	174.3	0.889
Vallisneria denseserrulata	Carotenoids	104.9	68.3	141.5	0.854

Note: Dose-response curves were fitted using a four-parameter log-logistic (LL.4) model. Model goodness-of-fit was assessed using the Pseudo-R^2. The 95% confidence intervals (CIs) were estimated via the delta method. The EC_{50} for Fv/Fm in *Vallisneria* (211.6 min/d) is an extrapolated value beyond the tested range (0–150 min/d) and is therefore not shown in Figure 3. Pseudo-R^2 values were rounded to three decimal places, and EC_{50} values and CIs were rounded to one decimal place for clarity.
