# Supplementary material for: Physiological Responses of Two Submerged Macrophytes to Pulsed Aerial Exposure: Contrasting Photoprotection and Oxidative Defense
Source: Plants (Basel). 2026 Oct 27;15(21):3327. doi: 10.3390/plants15213327 (PMC13765662; doi:10.3390/plants15213327)
Supplement: Supplementary file 1 [file plants-15-03327-s001.zip › Table S2 Rotated component matrix of PCA.pdf]

Table S2. Rotated component matrix of the principal component analysis (PCA).

Variable	PC1	PC2	PC3
Y(II)	0.098	**0.932**	-0.066
Y(NPQ)	0.038	**0.856**	0.234
Fv/Fm	0.205	**0.928**	0.264
Chl a	**0.956**	0.154	0.010
Chl b	**0.842**	0.079	0.037
Car	**0.907**	0.185	0.106
POD	**-0.890**	-0.170	0.314
SOD	**-0.606**	0.180	**0.680**
CAT	**-0.723**	0.146	**0.614**
MDA	0.148	0.192	**0.886**

Note: Extraction method: Principal component analysis. Rotation method: Varimax with Kaiser normalization. Rotation converged in 5 iterations. The first three principal components explained 87.14% of the total variance (PC1: 42.03%, PC2: 26.46%, PC3: 18.65%). Y(NO) was excluded from the PCA to avoid mathematical collinearity (since Y(II) + Y(NPQ) + Y(NO) = 1). Loadings with absolute values > 0.6 are highlighted in bold in the original analysis
